# Comparative Analysis of the Biochemical and Molecular Responses of *Nannochloropsis gaditana* to Nitrogen and Phosphorus Limitation: Phosphorus Limitation Enhances Carotenogenesis

**DOI:** 10.3390/md22120567

**Published:** 2024-12-18

**Authors:** Sun Young Kim, Hanbi Moon, Yong Min Kwon, Kyung Woo Kim, Jaoon Young Hwan Kim

**Affiliations:** National Marine Biodiversity Institute of Korea, Jangsan-ro 101-75, Seocheon 33662, Republic of Korea; dydn5588@naver.com (S.Y.K.); hbmoon@mabik.re.kr (H.M.); jichi9@mabik.re.kr (Y.M.K.); kimkw79@mabik.re.kr (K.W.K.)

**Keywords:** *Nannochloropsis gaditana*, biochemical response, molecular response, carotenoid, violaxanthin, RNA-seq

## Abstract

*Nannochloropsis gaditana* is well known for its potential for biofuel production due to its high lipid content. Numerous omics and biochemical studies have explored the overall molecular mechanisms underlying the responses of *Nannochloropsis* sp. to nutrient availability, primarily focusing on lipid metabolism. However, *N. gaditana* is able to synthesize other valuable products such as carotenoids, including violaxanthin, which has various biological functions and applications. In this study, we comparatively investigated the physiological, biochemical, and molecular responses of *N. gaditana* to nitrogen and phosphorus limitation, examining biomass production, photosynthetic activity, lipid, chlorophyll, and carotenoids content, and RNA-seq data. Nitrogen limitation decreased photosynthetic activity, chlorophyll content, and biomass production but increased lipid content. Phosphorus limitation substantially increased carotenoids content, with violaxanthin productivity of 10.24 mg/L, 3.38-fold greater than under the control condition, with little effect on biomass production or photosynthetic function. These results were generally consistent with the gene expression pattern observed in transcriptomic analysis. This integrated analysis shows that phosphorus limitation can be an economically competitive solution by enhancing valuable carotenoids while maintaining lipid and biomass production in *N. gaditana*.

## 1. Introduction

The increasing awareness of global warming, caused by high carbon dioxide (CO_2_) emissions and fossil fuel consumption, has triggered worldwide efforts to explore sustainable sources of energy and materials. Microalgae are recognized to play a crucial role in carbon neutralization through their photosynthetic ability. They have numerous advantages over plants, including rapid growth, high photosynthetic efficiency, no requirement for arable land, and limited seasonal variation [1,2,3,4]. They can synthesize valuable products, including lipids, proteins, carbohydrates, and pigments, using light energy, CO_2_, and inorganic nutrients. Therefore, microalgae can be considered light-driven cell factories for the conversion of CO_2_ into valuable bio-products [5].

More than 50,000 microalgal species are estimated to exist, of which around 30,000 have been identified [6,7]. It has also been reported that a number of microalgal species can be used for industrial applications in the food, feed, cosmetic, fertilizer, and energy sectors, including the green algae *Chlorella* sp., *Dunaliella salina*, and *Haematococcus pluvialis*, the diatoms *Phaeodactylum tricornutum*, the golden alga *Isochrysis galbana*, and the stramenopile alga *Nannochloropsis* sp. [8]. Among them, the small unicellular eustigmatophycean algae *Nannochloropsis* spp. are recognized as promising candidates for industrial biofuel production. They offer many desirable traits, including robust photoautotrophic growth at a commercial scale, tolerance to a wide range of culture conditions, high productivity of triacylglyceride (TAG) lipids, and stable transformation and gene expression along with the benefit of available sequenced haploid genomes and suitable genetic tools [9,10]. However, many challenges remain in terms of the economic viability of their cultivation solely for biofuel production at a commercial scale [11]. *Nannochloropsis* sp. can produce various valuable products, including eicosapentaenoic acid (EPA, an omega-3 fatty acid), beta-carotene, various xanthophylls (violaxanthin, vaucheriaxanthin, canthaxanthin, astaxanthin, neoxanthin, zeaxanthin, and antheraxanthin), phenolic compounds, and vitamins, which have various applications in the food, feed, cosmetic, and health care sectors [12]. Therefore, the coproduction of biofuels and high-value products through microalgal biorefineries has been proposed to improve the economic feasibility of microalgal utilization [13]. 

*Nannochloropsis* sp. contains violaxanthin and vaucheriaxanthin as primary pigments, which bind to violaxanthin-chlorophyll a binding protein (VCP) with chlorophyll a, to form the light-harvesting complex (LHC) for photosynthesis [10]. Violaxanthin reportedly has greater antioxidant activity than β-carotene and lutein [14]. It has also been demonstrated to have anti-inflammatory activity in murine macrophage cells [15], antiproliferative activity against human cancer cells [16], and protective effects against ultraviolet B-induced skin damage [17]. Due to the industrial potential of violaxanthin in the food, cosmetic, and pharmaceutical sectors, there has been a growing interest in increasing its production in *Nannochloropsis* sp. It has been reported that the pigment contents in *Nannochloropsis* sp. can be significantly altered by changing nutrients and culture conditions. Several studies have demonstrated the engineering of *Nannochloropsis* sp. to enhance violaxanthin production [18,19].

Microalgae have evolved the ability to change their biochemical composition in response to environmental stimuli such as light intensity, temperature, pH, and nutrient availability. Nitrogen (N) and phosphorus (P) are indispensable macronutrients required for microalgal growth. As microalgae are often exposed to conditions of limited N and P in the environment, they have evolved metabolic adaptations to cope with these nutrient restraints [20]. N is an essential component of proteins, nucleic acids (RNA and DNA), and chlorophylls. P is a constituent of nucleic acids, adenosine triphosphate (ATP), and phospholipids in the cellular membrane [21,22]. Therefore, the availability of N and P can substantially impact microalgal physiology and metabolism. N deficiency impairs cell growth, photosynthetic ability, and protein synthesis due to the degradation of N-containing compounds. N deprivation also increases amounts of neutral lipids, carbohydrates, and secondary carotenoids depending on the species [21,23,24]. P deprivation often induces changes in lipid classes, where P-containing lipids are replaced by non-phosphorus lipids, such as betaine lipids and glycolipids, to sustain cell membrane homeostasis [20]. Similarly to N deprivation, P deprivation also induces intracellular TAGs, but the TAG content may derive from the degradation of phospholipids rather than de novo fatty acid synthesis [20].

Numerous omics and biochemical studies have explored the overall molecular mechanisms of the response of *Nannochloropsis* sp. to N and P availability, with most focusing on lipid metabolism [20,22,24,25]. However, while *Nannochloropsis* sp. is well known for its high lipid productivity, it can also synthesize various valuable secondary metabolites, including carotenoids [26]. Further investigation is therefore vital to reveal the molecular mechanisms of carotenoid synthesis in response to nutrient availability and improve the economic feasibility of the utilization of *Nannochloropsis* sp. Previous studies have reported contrasting results regarding carotenoid synthesis in response to P deprivation. One study reported that phosphate-limited conditions increased the content of xanthophylls, including violaxanthin, zeaxanthin, and canthaxanthin [27]. However, another reported that most genes involved in carotenoid synthesis were transcriptionally downregulated under P deprivation conditions but did not directly measure carotenoid contents [24]. This discrepancy might be attributable to the difference in experimental conditions [28].

To address this controversial issue related to carotenogenesis in *Nannochloropsis* sp., in this study, we comparatively explored the physiological, biochemical, and molecular responses of *Nannochloropsis gaditana* to N-limited, P-limited, and control conditions, examining photosynthetic activity, biomass productivity, carotenoids content, fatty acid content, and transcriptomics. In contrast with the N-limited condition, the P-limited condition increased the content of carotenoids, including violaxanthin and vaucheriaxanthin, compared to the control condition with little effect on photosynthetic activity and biomass productivity. These results were consistent with the corresponding gene expression patterns determined by transcriptome sequencing. Our results support the modulation of the P supply in the culture of *N. gaditana* as a crucial and promising strategy to enhance the production of valuable carotenoids, including violaxanthin for economic viability.

## 2. Results and Discussion

### 2.1. Analysis of Cell Growth and Photosynthetic Activity Under N- and P-Limited Conditions

**Figure 1:**
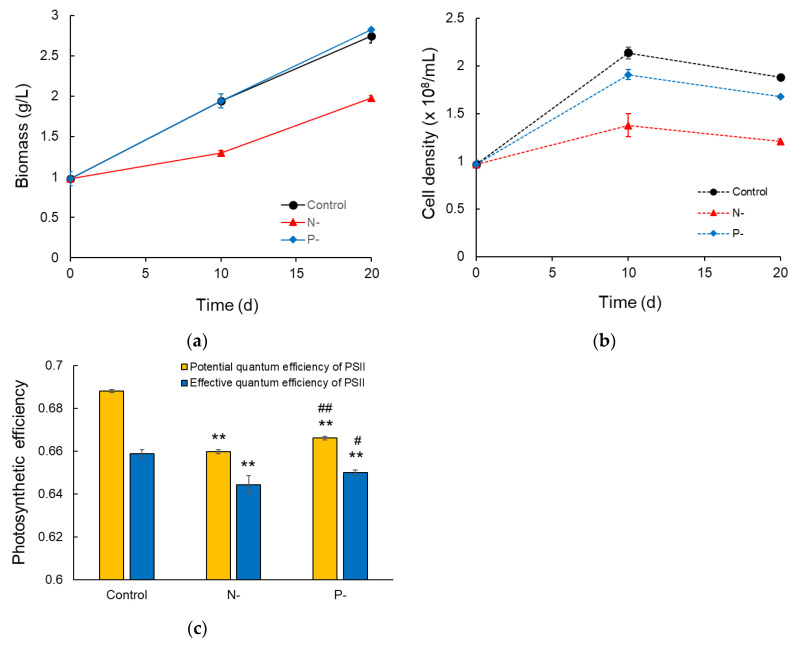
Cell growth and photosynthetic efficiency under the control, N-limited, and P-limited conditions. (**a**) Dry cell weight. (**b**) Cell density. Time denotes days after medium change for nutrient stress. Data and error bars are mean ± SD (*n* = 3). (**c**) Photosynthetic efficiency (potential quantum efficiency: F_v_/F_m_, effective quantum efficiency: YII, as described in methods). Data and error bars are mean ± SD (n = 4). ** denotes a *p*-value < 0.01 versus control condition (Student’s *t*-test). # and ## denote a *p*-value < 0.05 and a *p*-value < 0.01 versus N-limited condition, respectively (Student’s *t*-test).

The *N. gaditana* strain CCMP 526 was grown for 10 days before exposure to nutrient-stress conditions. Next, cells were grown in a medium containing 0.5% of either the N or P concentration of the control medium for nutrient limitation. N and P are the most important nutrients substantially impacting cell growth. Cell number, biomass, and photosynthetic efficiency were examined under N and P limitation conditions compared to the control condition to understand the cellular response of *N. gaditana* CCMP 526. Compared to the control condition, the cell number was 11% lower in the P-limited condition and 36% lower in the N-limited condition (Figure 1a). A previous study reported a greater reduction in the cell number under P-limited conditions [24], possibly due to its use of a P-free medium. Despite the decreased cell number compared to the control condition, the biomass was similar under the P-limited and control conditions throughout culturing, consistent with the previous report [24]. In contrast, the biomass was 33% and 28% lower under the N-limited condition than under the control condition at 10 and 20 days after the medium change, respectively (Figure 1b). These results indicate that cell weight was greater under the N- and P-limited conditions than under the control condition, as described previously [20,24].

The chlorophyll content per cell was largely lower under the N-limited condition than under the P-limited and control conditions (Table S1), as supported by the color of cultures (Figure S1); this is consistent with a previous study [20]. Given the change in chlorophyll content, photosynthetic activities of nutrient-limited conditions were analyzed using in vivo fluorescence measurements. The potential and effective quantum efficiencies of photosystem II (PSII) (F_v_/F_m_ and YII) were lower under the N-limited condition than under the P-limited and control conditions (Figure 1c). This result indicates that N deprivation negatively affects chlorophyll content and photosynthetic performance, thereby decreasing biomass productivity, which differs from the P-limited condition.

### 2.2. The Effects of Nutrient Limitation on Carotenoids and Lipids Productivity

**Figure 2:**
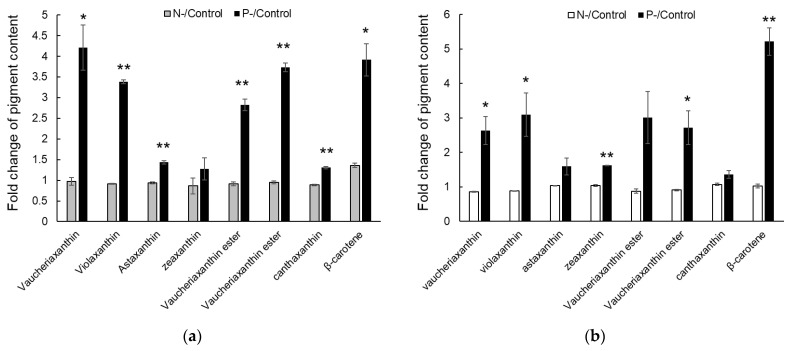
Relative carotenoid production under the N-limited and P-limited conditions compared to the control condition. (**a**) Fold change of carotenoid content (N-limited/control and P-limited/control) at 10 days after medium change. (**b**) Fold change of carotenoid content (N-limited/control and P-limited/control) at 20 days after medium change. Data and error bars are mean ± SD (n = 2). * and ** denote a *p*-value < 0.05 and a *p*-value < 0.01, respectively (Student’s *t*-test). Volumetric productions (mg/L) of each carotenoid are presented in Tables S2 and S3 of Supplementary Materials.

Carotenoid production in response to N- and P-limited conditions was determined and compared to the control condition. Interestingly, the production of all carotenoids was greater under the P-limited condition compared to the control condition at 10 and 20 days after the medium change. In contrast, most carotenoids showed similar or slightly reduced production under the N-limited condition compared to the control condition, except β-carotene at 10 days (Figure 2). Notably, in terms of volumetric production, violaxanthin was the most abundantly produced carotenoid in the P-limited condition (10.24 mg/L, 53.6% of all carotenoids), 3.38-fold greater than under the control condition (Tables S2 and S3). This was followed by vaucheriaxanthin and its ester form (5.55 mg/L), 3.1-fold greater than under the control condition, and astaxanthin (2.21 mg/L), 1.43-fold greater than under the control condition (Figure 2, Tables S2 and S3). These results suggest that P-limited conditions could be a promising strategy for enhancing the production of carotenoids, including violaxanthin, in *Nannochloropsis* sp. 

**Figure 3:**
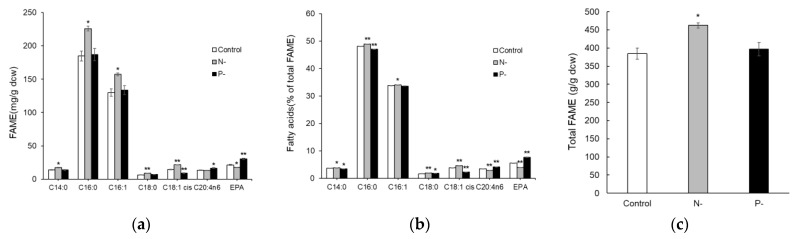
Lipid content under the control, N-limited, and P-limited conditions. (**a**) The profile of fatty acid content on the basis of dry cell weight. (**b**) Fatty acid composition (% of total FAME). (**c**) Total fatty acid content on the basis of dry cell weight. Data and error bars are mean ± SD (n = 2). * and ** denote a *p*-value < 0.05 and a *p*-value < 0.01 versus control condition (Student’s *t*-test).

In terms of lipid production, total fatty acid content was greatest under the N-limited condition and did not differ significantly between the P-limited and control conditions. The fatty acid profile showed that fatty acid composition was not substantially changed by nutrient limitation. The levels of C16:0 and C18:1 were slightly higher, while those of C20:4n6 and C20:5 were lower under the N-limited condition compared to the P-limited and control conditions (Figure 3).

### 2.3. Differential Gene Expression in Response to N and P Limitation

**Figure 4:**
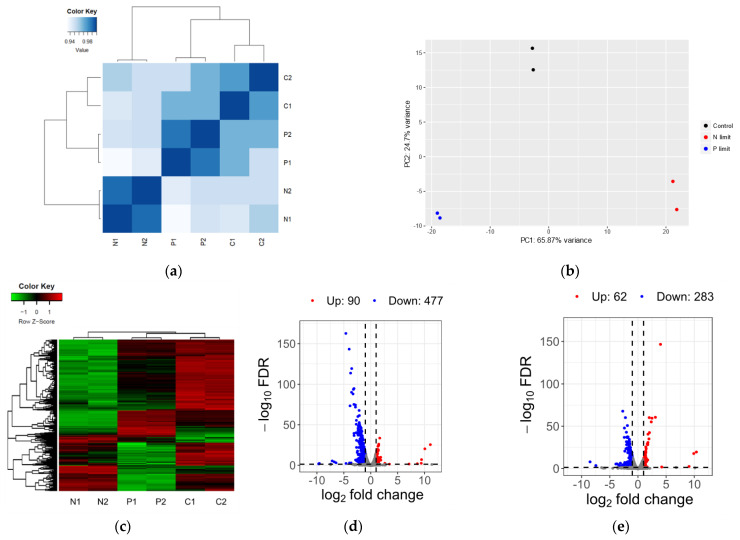
Global analysis of transcriptomes and DEGs. (**a**) Pearson’s correlation coefficient for transcriptomes between the control, N-limited, and P-limited conditions, each with two biological replicates. (**b**) Principal component analysis using PCAGO online software (https://pcago.bioinf.uni-jena.de/, accessed on 17 November 2024). (**c**) Heat map showing the expression level of DEGs in different samples. (**d**) Volcano plot of DEGs between the control and N-limited conditions. (**e**) Volcano plot of DEGs between the control and P-limited conditions. Red, blue, and gray points represent upregulated, downregulated, and nonregulated DEGs, respectively.

**Figure 5:**
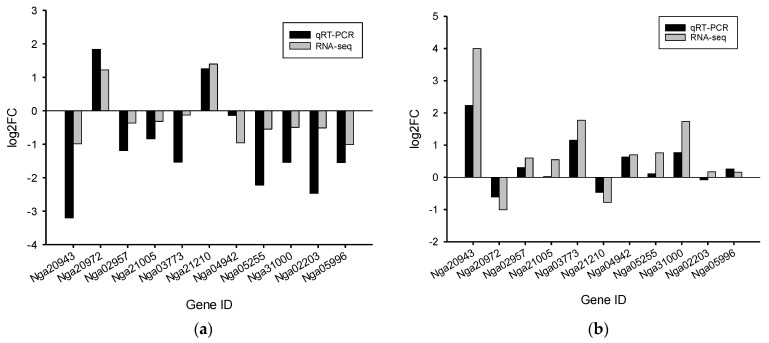
Validation of gene expression using qRT-PCR with three biological replicates. The transcript level of each gene under the (**a**) N-limited and (**b**) P-limited conditions was normalized to the level under the control condition and represented as log_2_ transformed value. Nga20943 (prolyl 4-hydroxylase); Nga20972 (ammonium transporter); Nga02957 (phytoene synthetase); Nga21005 (glyceraldehyde-3-phosphate dehydrogenase); Nga03773 (pyruvate decarboxylase); Nga21210 (glutaminase); Nga04942 (phosphoglucomutase); Nga05255 (phosphoglycerate kinase); Nga31000 (sodium phosphate symporter); Nga2203 (1-deoxy-D-xylulose-5-phosphate synthase); Nga05996 (phosphoglucose isomerase).

The transcriptome was analyzed using next-generation RNA sequencing (RNA-seq) to investigate the molecular basis of the response of *N. gaditana* to N and P limitation. Gene expression levels were compared between the control, N-limited, and P-limited conditions using culture samples collected at the same time point, as culture age can impact pigment production [26]. The transcriptomes were highly correlated within the same culture conditions, as evidenced by Pearson’s correlation coefficient (Figure 4a) and principal component analysis (Figure 4b). Transcriptome analysis revealed that 567 of 9052 total predicted genes exhibited significantly different expression levels under the N-limited condition compared to the control condition, of which 90 were upregulated and 477 were downregulated (Figure 4c,d). In contrast, 345 exhibited significantly different expression levels under the P-limited condition compared to the control condition, of which 62 were upregulated and 283 were downregulated (Figure 4c,e). These results indicate that P limitation has a weaker effect on the transcriptome than N limitation. Only 10 and 80 genes were upregulated and downregulated under both conditions, respectively, potentially representing essential genes regulated by nutrient limitation. In order to validate the gene expression patterns in the RNA-seq data, 11 genes were selected and analyzed using quantitative real-time PCR (qRT-PCR). The qRT-PCR results showed gene expression patterns similar to the RNA-seq results (Figure 5).

### 2.4. Impact of Nutrient Limitation on Genes Involved in Photosynthesis

Under the N-limited condition, transcriptomic analysis showed a substantial decrease in the expression of three genes in the chlorophyll biosynthetic pathway (Mg-chelatase subunit H, light-dependent NADPH:protochlorophyllide oxidoreductase, and Coproporphyrinogen III oxidase), which is consistent with the reduction in chlorophyll content. The gene encoding glutamyl-tRNA reductase, involved in the first step of the chlorophyll biosynthetic pathway, also showed slightly reduced expression. In addition, 19 genes encoding components of the LHC and 6 genes encoding components of PSII (PSII reaction center W, PSII oxygen-evolving enhancer protein 1, PSII oxygen-evolving complex protein, PSII 11 kDa protein, and extrinsic protein in PSII) were substantially downregulated, consistent with the reduced photosynthetic activity. The genes encoding the PSII stability/assembly factor, responsible for the de novo assembly and repair of PSII, also showed reduced expression, consistent with a previous study [20] (Table 1). In contrast with the N-limited condition, no substantial downregulation of photosynthesis-related genes was observed under the P-limited condition, supporting the evidence that P limitation has a weaker negative effect on the photosynthetic process than N limitation at the gene expression level and consistent with photosynthetic activity and chlorophyll content.

### 2.5. Impact of Nutrient Limitation on Genes Involved in Lipid Metabolism

The effect of nutrient limitation on the expression of genes involved in lipid metabolism was investigated to understand the molecular response in *N. gaditana*. While the N-limited condition increased total fatty acid methyl ester (FAME) content, there were no substantial changes in the overall expression of genes involved in the plastid fatty acid synthesis, as described in a previous study [20]. Regarding fatty acid synthesis, the gene encoding a plastid beta-ketoacyl-ACP synthase II involved in the condensation step of fatty acid biosynthesis (Naga_100002g173) was downregulated under the N-limited condition but upregulated in the P-limited condition. The gene encoding polyketide synthase (Naga_100093g2) was also significantly downregulated under the N-limited condition (Table S4).

TAG is biosynthesized in the endoplasmic reticulum, where an acyl-coenzyme A (CoA) group is added to glycerol-3-phosphate by the glycerol-3-phosphate acyltransferase (GPAT), producing lysophosphatidic acid. The gene encoding GPAT (Naga_100106g21) was downregulated under the N-limited condition. The last step in TAG synthesis, in which diacylglycerol (DAG) is converted into TAG, is catalyzed by diacylglycerol acyltransferase (DGAT). Among the 13 DGAT isoforms with one of DGAT1 family and 12 DGAT isoforms in the DGAT2 family [29], two DGAT2 isoforms were upregulated in each of the N-limited conditions (DGAT2a [Naga_100343g3], DGAT2b [Naga_100010g31]) and the P-limited conditions (DGAT2a [Naga_100343g3], DGAT2c [Naga_100251g8]) (Table S4). The expression of Lysophosphatidylglycerol acyltransferase (LPAT; Naga_100501g5), which catalyzes the conversion of lysophosphatidic acid into phosphatidic acid, did not change significantly under either N- or P-limited condition. As described previously [30], genes with the same function involved in the acyl-CoA-dependent TAG pathway were inconsistently expressed in *N. gaditana*. Multiple gene copies of the same enzymes may exist due to different cellular localizations. In addition, enzymes with multiple functions make it more challenging to understand the molecular mechanisms and regulation of the TAG synthesis pathway. Therefore, further investigation is required to reveal the unidentified genes and their functions involved in lipid metabolism.

In addition to de novo synthesis, TAGs can be synthesized via the acyl-CoA-independent pathway by remodeling membrane lipids. In this pathway, the phospholipid:diacylglycerol acyltransferase (PDAT) catalyzes the transfer of an acyl moiety from a phospholipid to DAG, forming TAG. However, one isoform of this gene (Naga_100004g173) in the genome of *N. gaditana* did not show significantly different expression under either N- or P-limited conditions. Fatty acids released from membrane lipids by lipases, including phospholipids and galactolipids, can be used to synthesize TAGs. Several identified lipases, including phospholipases and lysophospholipases, showed differential expression under N- and P-limited conditions (Table S4). 

In *Nannochloropsis* sp., EPA (C20:5) is synthesized as the major polyunsaturated fatty acid from the saturated fatty acid C18:0 through a serial enzymatic reaction by stearoyl-ACP desaturase, Δ9-, Δ12-, Δ6-, Δ5- and ω3-fatty acid desaturases (FAD), and a Δ6-fatty acid elongase [31]. Stearoyl-ACP desaturase (Naga_100013g52), Δ12-FAD (Naga_100092g4), and Δ9-FAD (Naga_100027g27) were upregulated under the P-limited condition, whereas ω3-FAD (Naga_100545g1) and Δ5-FAD (Naga_100273g7) were downregulated under the N-limited condition (Table S4), supporting evidence that EPA production was greater under the P-limited condition than under the N-limited and control conditions.

Diacylglyceryl-N,N,N-trimethylhomoserine (DGTS), a non-phosphorous and polar glycerolipid belonging to the betaine lipid group, is reportedly crucial for adaptation to phosphate deficiency in *Nannochloropsis* sp. [32]. In eukaryotes, betaine lipid synthase 1 is responsible for the entire DGTS biosynthesis process [33]. DGTS is highly enriched with eicosapentaenoic acid (EPA) and DGTS content increases by P starvation [32,34]. It has also been reported that sulfoquinovosyldiacylglycerol synthesis becomes crucial when phosphate is limited [35]. In our study, three genes encoding UDP-glucose pyrophosphorylase (Naga_100013g89, Naga_100003g178, Naga_100071g9) and one gene encoding phosphoglucomutase (Naga_100065g10), each involved in the synthesis of sulfoquinovosyldiacylglycerol, were upregulated under the P-limited condition. In contrast, two genes encoding UDP-glucose pyrophosphorylase (Naga_100013g89 and Naga_100071g9) and one gene encoding UDP-sulfoquinovose synthase (Naga_100038g9) were downregulated under the N-limited condition. The gene encoding DGTS synthase (Naga_100016g36) was upregulated under the P-limited condition. In addition, the gene encoding glycerophosphodiester phosphodiesterase (Naga_100131g11) was upregulated under the P-limited condition (Table S4); this gene is involved in the hydrolysis of the intermediate products of phospholipid catabolism, glycerophosphodiesters, into glycerol-3-phosphate, a precursor of phosphate [36]. These results are consistent with previous studies on membrane lipids recycling associated with P deprivation, where phospholipids are substituted with non-P lipids such as betaine lipids or glycolipids [22,37]. This also supports evidence that thylakoid membrane integrity is largely maintained under P limitation due to membrane lipid remodeling, resulting in a weaker impact on photosynthetic activity than under N limitation.

### 2.6. Impact of Nutrient Limitation on Genes Involved in Central Carbon Metabolism

*N. gaditana* synthesizes chrysolaminarin or laminarin, glucans with various ratios of β-(1,3) to β-(1,6)-linked glucose, as the primary storage carbohydrate. This differs from green algae, which accumulate starch as the primary storage carbohydrate. The synthesis of β-1,3-glucan is catalyzed by UDP-glucose pyrophosphorylase followed by β-1,3-glucan synthase [24]. Three genes encoding UDP-glucose pyrophosphorylase in *N. gaditana* (Naga_100013g89, Naga_100003g178, and Naga_100071g9) showed slightly decreased expression under the N-limited condition but increased expression under the P-limited condition compared to the control condition. The expression of β-1,3-glucan synthase, encoded by a single-copy gene, increased slightly under the N-limited condition. Glucokinase (GK), phosphofructokinase (PFK), and pyruvate kinase (PK) control glycolysis pathway flux by catalyzing irreversible reactions [24]. Two GK genes, two PFK genes, and five PK genes have been identified in *N. gaditana*. GK and PFK showed slightly increased expression under the N-limited condition compared to the control condition, while PK showed similar expression. The overall transcripts of each reaction showed slightly increased expression under the P-limited condition compared to the control condition. The expression of the pyruvate decarboxylase (PDC) gene was substantially increased under the P-limited condition compared to the control condition (log_2_ Fold Change = 1.77, FDR = 1.21 × 10^−31^), indicating that the pyruvate dehydrogenase complex (PDHC) bypass pathway was preferred to the PDHC pathway to provide acetyl-CoA for de novo fatty acid synthesis under the P-limited condition, as described previously [24]. 

### 2.7. Impact of Nutrient Limitation on Genes Involved in Carotenoid Biosynthesis

**Figure 6:**
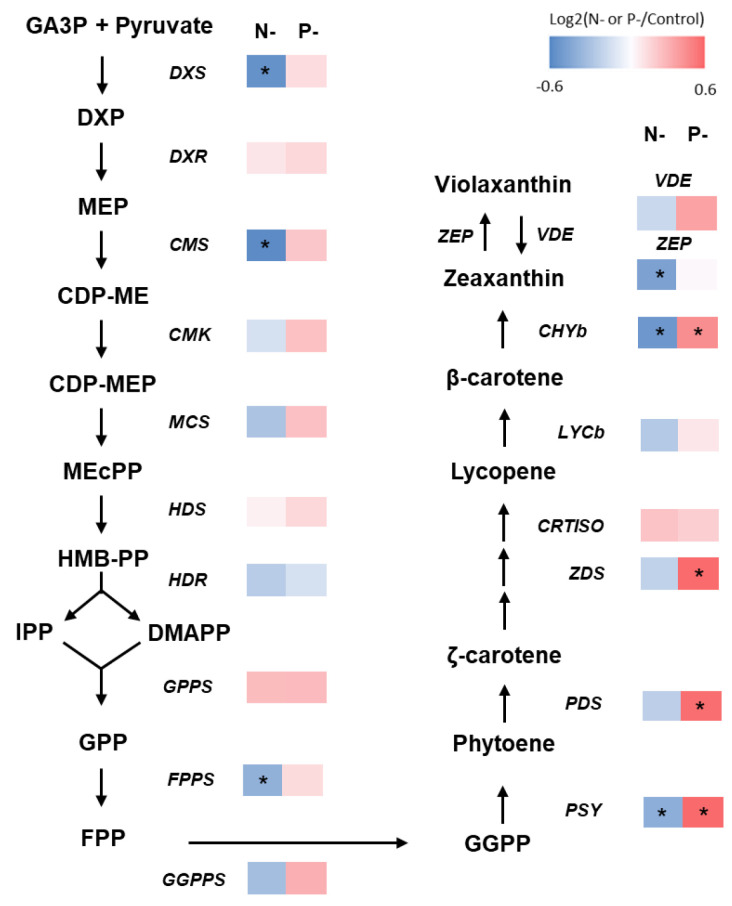
Comparative analysis of gene expression (RNA-seq) involved in MEP and carotenogenesis pathway. The expression level of each gene under the N-limited and P-limited conditions was normalized to the level under the control condition and represented as log_2_ transformed value. Heat map shows the log_2_(fold change) values. Asterisk indicates false discovery rate (FDR) adjusted *p*-value < 0.05.

It has been reported that carotenoids are synthesized from the 5-carbon isoprenoids precursors isopentenyl pyrophosphate (IPP) and dimethylallyl pyrophosphate (DMAPP) from the chloroplastic methylerythritol phosphate (MEP) pathway, rather than the cytosolic mevalonate (MVA) pathway in *Nannochloropsis* sp. [24]. Most genes in the MEP and carotenogenesis pathways, including 1-deoxy-D-xylulose-5-phosphate synthase (DXS), 2-c-methyl-d-erythritol 4-phosphate cytidylyltransferase (CMS), farnesyl diphosphate synthase (FPPS), and phytoene synthetase (PDS), showed decreased expression under the N-limited condition compared to the control condition. In contrast, most genes in the MEP and carotenogenesis pathways, including phytoene synthetase (PSY), phytoene desaturase (PDS), and zeta-carotene desaturase (ZDS), showed increased expression under the P-limited condition compared to the control condition. While the degree of upregulation and downregulation in these cases was small, the overall expression of genes involved in the MEP and carotenogenesis pathway correlates with carotenoid production under N- and P-limited conditions (Figure 6). Therefore, these results support that P limitation is a feasible strategy to enhance the production of carotenoids, including violaxanthin, in *N. gaditana*. The results differ from a previous study, which showed that genes involved in the MEP pathway and carotenogenesis were downregulated under P deprivation [24]. This difference might be due to experimental design and set-ups regarding the level of nutrient withdrawal, the treatment duration, and culture conditions, as described previously [28].

## 3. Materials and Methods

### 3.1. Cell Culture Conditions

*N. gaditana* CCMP 526 was obtained from the National Center for Marine Algae and Microbiota (East Boothbay, ME, USA). The cells were grown in a CO_2_ incubator at 25 °C and 0.5% CO_2_ under continuous light at a light intensity of 50 μmol photons/m^2^/s. The cultures were inoculated with an initial optical density at 800 nm (OD_800_) of 0.2. The cells were grown in a modified F/2 medium enriched with N (150 mg/L NaNO_3_) and P (11.3 mg/L NaH_2_PO_4_) for 10 days. Next, they were harvested and washed twice with medium: the modified F/2 medium, F/2 medium containing 0.75 mg/L NaNO_3_ and 11.3 mg/L NaH_2_PO_4_, and F/2 medium containing 150 mg/L NaNO_3_ and 0.0565 mg/L NaH_2_PO_4_ for the control, N-limited, and P-limited cultures, respectively. The cells were then resuspended in the corresponding medium and grown under the same conditions described above to investigate their response of cells to nutrient limitation.

### 3.2. Measurement of Growth Parameters

The cell number of *N. gaditana* CCMP 526 was determined by counting using a hemocytometer under light microscopy. Biomass was determined by measuring dry cell weight, as described previously [38]. The photosynthetic efficiency parameters were evaluated by measuring the in vivo chlorophyll fluorescence of dark-adapted cells for 30 min using a chlorophyll fluorometer (FMS2, Hansatech, UK). The parameters maximum photosynthetic F_v_/F_m_ was calculated as (F_m_ − F_0_)/F_0_. The quantum yield of PSII (YII) was calculated as (F_m_′ − F_s_)/F_m_′.

### 3.3. Analysis of Pigments and Lipids Contents

For analysis of pigment contents, 5 mL of cell culture was harvested with centrifugation at 5000× *g* for 15 min, and the cell pellet was stored at −80 °C. Next, the frozen cell pellet was extracted with 5 mL ethanol by vortexing at maximum speed for 15 min twice at room temperature. The extract was filtered using a polytetrafluoroethylene syringe filter (pore size of 0.2 μm) to remove cell debris. The chlorophyll content was determined spectrophotometrically as previously described [39]. Carotenoid content was analyzed using a high-performance liquid chromatography (HPLC) system (1260 infinity, Agilent, Santa Clara, CA, USA) with a Horizon C18/PFP column (150 mm × 4.6 mm, and 3 μm particle size; Horizon Chromatography, Halifax, UK) as previously described [40]. Carotenoids were detected using diode-array spectroscopy (300–720 nm). Liquid chromatography-mass spectrometry (LC-MS) analysis was conducted using an Agilent 1290 infinity II HPLC system with a diode array detector and ISQ EC mass spectrometer (Thermo Fisher Scientific, Waltham, MA, USA) with a HESI-II electrospray ionization source. Carotenoids were separated using a Horizon C18/PFP column (150 mm × 4.6 mm and 3 μm particle size), as described above, except that 0.1% (*v*/*v*) formic acid was added to the mobile phase solvents. The MS analysis was performed using the following parameters: vaporizer temperature = 317 °C; ion transfer tube temperature = 350 °C; optimum sheath gas pressure = 58.8 psig; auxiliary gas pressure = 5.2 psig; sweep gas pressure = 2 psig; and positive mode with a source voltage of 4 kV and variable collision-induced dissociation (CID) voltage. Identification was performed based on the comparison of the retention times and UV-visible and mass spectral characteristics of peaks to those of standards and data available in the literature [41,42]. The pigments were quantified using calibration curves of standard pigments (Sigma-Aldrich, St. Louis, MO, USA) or by comparing their peak area with that of violaxanthin.

Lipids were extracted from cell culture using a modified Bligh−Dyer method as previously described [43]. Briefly, 3 mL of chloroform/methanol (1:2 *v*/*v*) was added to 0.8 mL of liquid culture in a 15 mL Teflon screw-capped glass tube, and the mixture was intermittently vortexed at room temperature for 30 min. After adding 1 mL of chloroform and water, the mixture was vortexed for 1 min and centrifuged for 10 min at 2000 rpm. The lower phase was collected using a Pasteur pipet, and chloroform was evaporated under a stream of nitrogen gas. 

Fatty acids were analyzed with gas chromatography after the acid-catalyzed transesterification of total lipid extracts as previously described [43]. Briefly, 2 mL of methanolic sulfuric acid (3%, *v*/*v*) was added to a 15 mL, screw-capped, glass tube containing the total lipid extract in 1 mL of hexane. The mixture was vortexed and heated at 95 °C for 1.5 h. After cooling, 2 mL of water and hexane were added, and FAMEs were separated by collecting the organic phase. The extracted FAMEs were analyzed using a gas chromatograph (7890A GC, Agilent, Santa Clara, CA, USA) equipped with a flame ionization detector and a DB-FastFAME column (30 m × 0.25 mm, and 0.25 µm; Agilent, Santa Clara, CA, USA) with the following conditions: injection volume 1 μL; split ratio 1:50; injector temperature 250 °C; detector temperature 280 °C; and oven temperature held at 50 °C for 0.5 min, increased to 194 °C at 30 °C/min, and increased to 240 °C at 5 °C/min. The FAMEs were identified and quantified by retention time and comparison with Supelco 37 Component FAME Mix (Sigma-Aldrich, St. Louis, MO, USA).

### 3.4. RNA Extraction and RNA-Seq Analysis

Cell cultures were harvested for RNA-seq analysis 5 days after changing the medium from the modified F/2 medium to the control, N-, and P-limited medium. RNAprotect Cell Reagent (Qiagen, Hilden, Germany) was added to the cell culture according to the manufacturer’s instructions. After incubation the mixture for 5 min at room temperature, total RNA was extracted from the cell pellets using a TRIzol Plus RNA Purification Kit (Invitrogen, Carlsbad, CA, USA) according to the manufacturer’s instructions. Contaminating genomic DNA in total RNA extracts was removed using Turbo DNase (Invitrogen), and total RNA was purified using a PureLink RNA Mini Kit (Invitrogen). RNA quality and concentration were assessed using a Bioanalyzer 2100 (Agilent Technologies, Santa Clara, CA, USA) and a NanoDrop 8000 (Thermo Scientific, Waltham, MA, USA). Messenger RNA (mRNA) was purified and fragmented from total RNA using poly-T oligo-attached magnetic beads with two rounds of purification. The mRNA sequencing libraries were prepared using an Illumina Truseq stranded mRNA library prep kit (Illumina, San Diego, CA, USA) according to the manufacturer’s instructions. RNA sequencing was performed with paired-end 100 bp reads on an Illumina NovaSeq 6000 system (DNA LINK, Inc., Seoul, Republic of Korea). The reads were aligned to the reference transcriptome of *N. gaditana* CCMP 526 using Kallisto (version 0.43.1) [44]. Genes with low expression levels were excluded using the filterByExpr function of the edgeR package (version 3.34.1) in the R statistical software (version 4.1.3). All gene counts were adjusted to counts per million (CPM) reads and normalized using the trimmed mean of M values (TMM) method. The gene expression patterns under the N- and P-limited conditions were compared with those under the control culture condition as the reference. Differentially expressed genes (DEGs) were identified among libraries using the exactTest function of the edgeR package in R software (version 4.1.3). Genes with a false discovery rate (FDR) adjusted *p*-value < 0.05 and |log2fc| ≥ 1.0 (|fold change of |≥ 2) were considered DEGs. The PCA analysis was performed using the PCAGO online software (“https://pcago.bioinf.uni-jena.de/ (accessed on 16 November 2024)”) as described [45].

### 3.5. Quantitative Real-Time PCR

First-strand complementary DNA (cDNA) was synthesized from 500 ng of total RNA obtained as described above using the SuperiorScript III RT Master Mix (Enzynomics, Daejeon, Republic of Korea) according to the manufacturer’s instructions. Next, qRT-PCR was performed with gene specific primers (Table S5) and TOPreal SYBR Green qPCR premix (Enzynomics) using a CFX Connect Real-Time PCR Detection System (Bio-Rad, Hercules, CA, USA) under the following conditions: Initial denaturation at 95 °C for 12 min, 40 cycles of 95 °C for 10 s, 60 °C for 15 s, and 72 °C for 30 s. The melting curves were generated by increasing the temperature by 0.5 °C every 5 s from 65 °C to 95 °C. The cycle threshold value (CT) and differential expression were calculated using the 2^−Δ Δ CT^ method [46], with β-actin used as a housekeeping gene to normalize gene expression. 

### 3.6. Statistical Analysis

Data were represented as means ± SD. All statistical analyses were performed using Student’s *t*-test in Excel, and *p* < 0.05 was considered statistically significant.

## 4. Conclusions

*N. gaditana* has great potential for producing biofuels due to its high lipid contents, but its cultivation for this purpose alone is economically unattractive. However, it can also produce various carotenoids, including violaxanthin, which have various biological functions and industrial applications. Therefore, the coproduction of biofuels and valuable carotenoids could be a competitive solution for the commercialization of *N. gaditana*. Because growth conditions affect the biochemical contents of microalgal cells, cultivation parameters such as light intensity, temperature, and nutrient environment must be optimized. Numerous biochemical and omics studies have explored the mechanisms of the response of *Nannochloropsis* sp. to nutrient availability. However, most have focused on lipid metabolism. Therefore, in this study, we comparatively analyzed the physiological, biochemical, and molecular responses of *N. gaditana* to N and P limitation. N limitation decreased chlorophyll content and photosynthetic activity but increased lipid content, concomitant with reduced biomass production. P limitation substantially improved carotenoid content compared to the control and N-limited conditions but did not significantly affect biomass production or photosynthetic function. The volumetric production of violaxanthin was 10.24 mg/L under the P-limited condition, 3.38-fold greater than under the control condition. The physiological and biochemical responses of *N. gaditana* were generally consistent with the gene expression patterns determined with RNA-seq. Our results indicate that P limitation could be a strategy to increase violaxanthin production without compromising biomass production, making it a promising approach to improve the economic viability of *N. gaditana* cultivation.

## Figures and Tables

**Table 1:** Gene expression related to photosynthesis in nitrogen-limited condition.

Gene	Function	log_2_FC (N-/Control)	FDR
NgLHCf1 (Naga_100012g50)	Light harvesting complex	−3.09	1.72 × 10^−37^
NgLHCf2 (Naga_100005g99)	Light harvesting complex	−1.79	2.49 × 10^−27^
NgLHCf3 (Naga_100157g5)	Light harvesting complex	−1.64	8.49 × 10^−20^
NgLHCf4 (Naga_100168g14)	Light harvesting complex	−2.15	2.15 × 10^−40^
NgLHCf5 (Naga_100017g59)	Light harvesting complex	−2.36	2.73 × 10^−38^
NgLHCf6 (Naga_100004g86)	Light harvesting complex	−2.04	1.51 × 10^−31^
NgLHCf7 (Naga_100013g28)	Light harvesting complex	−1.86	6.00 × 10^−25^
NgLHCf8 (Naga_100027g19)	Light harvesting complex	−2.03	1.85 × 10^−30^
NgLHCr1 (Naga_100002g18)	Light harvesting complex	−1.84	3.50 × 10^−25^
NgLHCr2 (Naga_100168g13)	Light harvesting complex	−2.02	6.80 × 10^−29^
NgLHCr3 (Naga_100017g83)	Light harvesting complex	−2.69	1.61 × 10^−53^
NgLHCr4 (Naga_100092g17)	Light harvesting complex	−1.07	5.21 × 10^−11^
NgLHCr5 (Naga_100434g4)	Light harvesting complex	−2.67	1.10 × 10^−58^
NgLHCr6 (Naga_100641g3)	Light harvesting complex	−2.23	1.68 × 10^−40^
NgLHCx1 (Naga_100173g12)	Light harvesting complex	−2.03	6.29 × 10^−29^
psbW (Naga_100040g31)	PS II reaction center W	−0.94	9.75 × 10^−06^
psbO (Naga_100313g2)	PS II O_2_-evolving enhancer protein	−0.95	1.32 × 10^−07^
psbP (Naga_100119g18)	PS II O_2_-evolving complex protein	−0.82	4.26 × 10^−07^
Pbs27 (Naga_100005g25)	PS II 11 kDa protein	−1.34	1.93 × 10^−17^
psbQ (Naga_100273g6)	extrinsic protein in PS II	−1.66	3.19 × 10^−24^
psbU (Naga_100076g3)	PS II 12 kDa extrinsic protein	−0.79	4.73 × 10^−05^
CPOX (Naga_100665g2)	Coproporphyrinogen III oxidase	−0.83	4.47 × 10^−08^
CHLH (Naga_100105g7)	Mg-chelatase subunit H	−0.99	7.23 × 10^−10^
POR (Naga_100258g4)	Light-dependent NADPH:protochlorophyllide oxidoreductase	−0.74	8.98 × 10^−07^
Ycf48 (Naga_100114g2)	PS II stability/assembly factor	−0.51	6.77 × 10^−04^

## Data Availability

The data used to support the findings of this study are available from the corresponding author upon request.

## Supplementary Materials

The following supporting information can be downloaded at https://www.mdpi.com/article/10.3390/md22120567/s1. Table S1: Chlorophyll contents of *N. gaditana* under different culture conditions. Figure S1: Images of *N. gaditana* cultures under different culture conditions. Table S2: Volumetric carotenoid productivity (mg/L) at 10 days. Table S3: Volumetric carotenoid productivity (mg/L) at 20 days. Table S4: Responses of genes involved in lipid metabolism to nitrogen and phosphorus limitation. Table S5: Primers used for qRT-PCR.
