# Supplementary material for: Comparative Analysis of the Biochemical and Molecular Responses of Nannochloropsis gaditana to Nitrogen and Phosphorus Limitation: Phosphorus Limitation Enhances Carotenogenesis
Source: Mar Drugs. 2024 Dec 18;22(12):567. doi: 10.3390/md22120567 (PMC11677669; doi:10.3390/md22120567)
Supplement: Supplementary file 1 [file marinedrugs-22-00567-s001.zip › marinedrugs-3348150-supplementary.pdf]

Supplementary Materials

Comparative analysis of the biochemical and molecular responses of *Nannochloropsis gaditana* to nitrogen and phosphorus limitation: Phosphorus limitation enhances carotenogenesis

Sun Young Kim, Hanbi Moon, Yong Min Kwon, Kyung Woo Kim and Jaoon Young Hwan Kim

Table S1. Chlorophyll contents of *N. gaditana* under different culture conditions.

	Control	N-limited	P-limited
Chlorophyll (μg/mL)	4.10 ± 0. 22	1.89 ± 0.06	3.87 ± 0.01
Chlorophyll (pg/cell)	0.022 ± 0.001	0.016 ±0.0001	0.023 ±0.0003

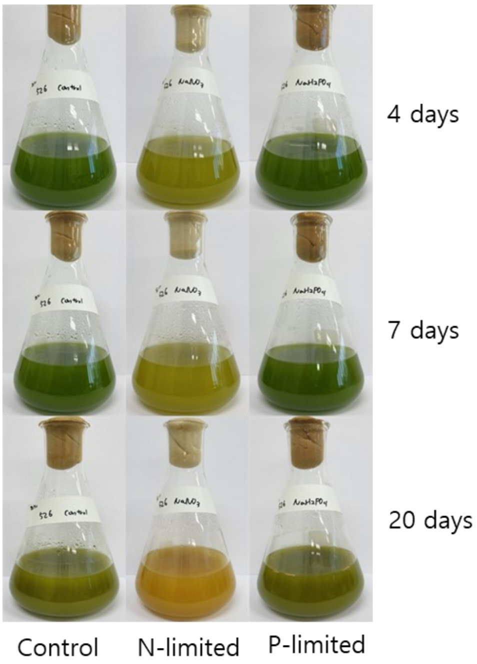

Figure S1. Images of *N. gaditana* cultures under different culture conditions. The image shows the culture of *N. gaditana* at 4, 7, 20 days after change of each medium (Control, N-limited, P-limited medium).

Table S2. Volumetric carotenoid productivity (mg/L) at 10 days after medium change.

Carotenoid (mg/L)	Control	N-limited	P-limited
Vaucheriaxanthin	0.18±0.03	0.18±0.01	0.76±0.01
Violaxanthin	3.03±0.14	2.76±0.08	10.24±0.32
Astaxanthin	1.54±0.09	1.44±0.04	2.21±0.06
zeaxanthin	0.44±0.07	0.37±0.02	0.55±0.03
Vaucheriaxanthin ester	1.31±0.10	1.19±0.03	3.69±0.08
Vaucheriaxanthin ester	0.30±0.02	0.28±0.00	1.10±0.03
canthaxanthin	0.19±0.01	0.16±0.00	0.24±0.00
β-carotene	0.08±0.03	0.11±0.04	0.33±0.14
sum	7.06±0.33	6.50±0.23	19.12±0.69

Table S3. Volumetric carotenoid productivity (mg/L) at 20 days after medium change.

Carotenoid	Control	N-limited	P-limited
Vaucheriaxanthin	0.13±0.01	0.11±0.00	0.34±0.07
Violaxanthin	1.97±0.06	1.74±0.07	6.13±1.41
Astaxanthin	1.76±0.08	1.82±0.08	2.82±0.56
zeaxanthin	0.33±0.07	0.35±0.06	0.54±0.11
Vaucheriaxanthin ester	0.77±0.00	0.68±0.05	2.32±0.58
Vaucheriaxanthin ester	0.19±0.01	0.17±0.01	0.52±0.12
canthaxanthin	0.22±0.01	0.23±0.01	0.30±0.03
β-carotene	0.04±0.01	0.04±0.01	0.23±0.09
sum	5.41±0.24	5.15±0.31	13.19±2.96

Table S4. Responses of genes involved in lipid metabolism to nitrogen and phosphorus limitation.

Condition	Gene	Function	log_2FC (N- or P-/Control)	FDR
N-	KASII (Naga_100012g50)	beta-ketoacyl-ACP synthase II	-0.54	6.38E-04
N-	FASI (Naga_100093g2)	Polyketide synthase	-1.28	2.34E-04
N-	ω3-FAD (Naga_100545g1)	omega-3 fatty acid desaturase	-0.58	9.49E-05
N-	Δ5-FAD (Naga_100273g7)	delta 5 fatty acid desaturase	-0.50	0.012
N-	Naga_100241g4	Lipase	-0.47	2.52E-03
N-	Naga_100454g3	phospholipase d	-1.06	2.98E-04
N-	Naga_100043g24	Lipase, class 3	-0.74	1.25E-03
N-	Naga_100009g65	group XIIA secretory phospholipase A2-like	1.37	3.36E-19
N-	UGPase (Naga_100071g9)	UDP-glucose pyrophosphorylase	-0.92	2.78E-06
N-	SQD1 (Naga_100038g9)	UDP-sulfoquinovose synthase	-0.50	8.12E-04
N-	DGAT(Naga_100343g3)	diacylglycerol o-acyltransferase 2	0.52	4.68E-04
N-	DGAT(Naga_100010g31)	diacylglycerol o-acyltransferase 2	0.55	1.07E-04
N-	Naga_100040g9	Acyl transferase/acyl hydrolase/lysophospholipase	0.63	0.035
P-	KASII (Naga_100012g50)	beta-ketoacyl-ACP synthase II	0.43	7.78E-03
P-	GPAT (Naga_100106g21)	glycerol-3-phosphate o-acyltransferase	-0.45	2.35E-03
P-	DGAT(Naga_100343g3)	diacylglycerol o-acyltransferase 2	0.59	5.82 E-05
P-	DGAT(Naga_100251g8)	diacylglycerol o-acyltransferase 2	0.49	1.73E-03
P-	Naga_100009g65	group XIIA secretory phospholipase A2-like	1.39	2.58E-19
P-	Naga_100454g3	phospholipase d	-0.75	0.015
P-	Naga_100040g9	Acyl transferase/acyl hydrolase/lysophospholipase	1.53	5.96E-09
P-	FAD(Naga_100013g52)	stearoyl-ACP desaturase	0.53	2.59E-03
P-	Δ12-FAD (Naga_100092g4)	delta-12 fatty acid desaturase	1.27	1.74E-07
P-	Δ9-FAD (Naga_100027g27)	delta-9 fatty acid desaturase	0.87	1.11E-05
P-	UGPase (Naga_100013g89)	UDP-glucose pyrophosphorylase	0.34	0.019
P-	UGPase (Naga_100003g178)	UDP-glucose pyrophosphorylase	0.58	2.87E-05
P-	UGPase (Naga_100071g9)	UDP-glucose pyrophosphorylase	0.44	0.042
P-	PGM (Naga_100065g10)	phosphoglucomutase	0.62	9.98E-04
P-	BTA1L (Naga_100016g36)	DGTS synthase	1.05	1.15E-12
P-	GDPD (Naga_100131g11)	glycerophosphoryl diester phosphodiesterase	1.66	1.13E-29

Table S5. Primers used for qRT-PCR.

Gene	Forward primer	Reverse primer
Nga20943 (prolyl 4-hydroxylase)	ATGAGCTGCAACCCTCTACC	ATGTCGCGCCAGATATTCCA
Nga20972 (ammonium transporter)	CCACCCTCCTCATGTCCTTC	GCTAGGGGAATGGTGTTGGA
Nga02957 (phytoene synthetase)	GTCGATACCAGTCGGAGCAA	AACGTCTGGAGGAATCGCAT
Nga21005 (glyceraldehyde-3-phosphate dehydrogenase)	CACAAACTGCGGGTTGAGAG	CACAACCTACGAGGCCATCT
Nga03773 (pyruvate decarboxylase)	GGTCTTCCTGGAGCTCATCC	CTCATTCAGGGGCCACATCT
Nga21210 (glutaminase)	TTTAAGCATGACCCTGCGGA	AGCGTCAAGGACATCTCCAG
Nga04942 (phosphoglucomutase)	ACAACATGCAAAACGGGGAG	CACCTCCACCACCATCTCTC
Nga05255 (phosphoglycerate kinase)	CCACCATTGAGTACCTGGCT	TCTCCAATGCAGTCCTCCAC
Nga31000 (sodium phosphate symporter)	TATACCGAGGGTTCAACGGC	CCAGGAGAGGACGATAGGGA
Nga2203(1-deoxy-D-xylulose-5-phosphate synthase)	GACCGAAAAGGGGTACGGAT	GTTGGCAAATGTCGTGGTCA
Nga05996 (phosphoglucose isomerase)	ATGTCTTTCCTGGGCTACCG	GGAAGAAAGAATGCTGGCCG
